# Insight into SARS-CoV-2 Omicron variant immune escape possibility and variant independent potential therapeutic opportunities

**DOI:** 10.1016/j.heliyon.2023.e13285

**Published:** 2023-01-31

**Authors:** Mohammad Shah Alam

**Affiliations:** Department of Anatomy and Histology, Bangabandhu Sheikh Mujibur Rahman Agricultural University, Gazipur 1706, Bangladesh

**Keywords:** Omicron, Immune escape, Variants, Humoral immunity, T cell immunity

## Abstract

The Omicron, the latest variant of severe acute respiratory syndrome coronavirus 2 (SARS-CoV-2), was first detected in November 2021 in Botswana, South Africa. Compared to other variants of SARS-CoV-2, the Omicron is the most highly mutated, with 50 mutations throughout the genome, most of which are in the spike (S) protein. These mutations may help the Omicron to evade host immunity against the vaccine. Epidemiological studies suggest that Omicron is highly infectious and spreads rapidly, but causes significantly less severe disease than the wild‐type strain and the other variants of SARS-CoV-2. With the increased transmissibility and a higher rate of re-infection, Omicron has now become a dominant variant worldwide and is predicted to be able to evade vaccine-induced immunity. Several clinical studies using plasma samples from individuals receiving two doses of US Food and Drugs Administration (FDA)-approved COVID-19 vaccines have shown reduced humoral immune response against Omicron infection, but T cell-mediated immunity was well preserved. In fact, T cell-mediated immunity protects against severe disease, and thus the disease caused by Omicron remains mild. In this review, I surveyed the current status of Omicron variant mutations and mechanisms of immune response in the context of immune escape from COVID-19 vaccines. I also discuss the potential implications of therapeutic opportunities that are independent of SARS-CoV-2 variants, including Omicron. A better understanding of vaccine-induced immune responses and variant-independent therapeutic interventions that include potent antiviral, antioxidant, and anti-cytokine activities may pave the way to reducing Omicron-related COVID-19 complications, severity, and mortality. Collectively, these insights point to potential research gaps and will aid in the development of new-generation COVID-19 vaccines and antiviral drugs to combat Omicron, its sublineages, or upcoming new variants of SARS-CoV-2.

## Highlights

• The Omicron is the most highly mutated variant compared to other variants of SARS-CoV-2.
• Omicron is highly infectious and spreads rapidly, but causes less severe disease than other variants.
• Omicron is now a dominant variant worldwide and is predicted to be able to escape immunity.
• Humoral immunity from vaccines was found to reduced, but T cell immunity was still protective against Omicron infection.
• Drugs targeting oxidative stress, cytokines, and viral replicase exhibit beneficial effects in Omicron-infected patients.

## 1 Introduction

A deadly ongoing coronavirus disease 2019 (COVID-19) pandemic caused by severe acute respiratory syndrome coronavirus-2 (SARS-CoV-2) presented a devastating global health crisis [1,2]. As of November 2022, COVID-19 has caused more than 640 million infections and 6 million deaths worldwide [3]. Despite an estimated 69% of the world's population receiving the COVID-19 vaccine (at least one dose), the disease is still being transmitted and the pandemic is far from over [4]. The SARS-CoV-2 virus has mutated over time, evolving into different variants and evading antibodies to infect more people. The fifth and latest variant of SARS-CoV-2, Omicron (B.1.1.529.1 or BA.1) was first identified on November 24, 2021 by the World Health Organization (WHO) from samples collected in Botswana and South Africa on 11 and November 14, 2021 [5]. Before the emergence of this variant, we also experienced Alpha, Beta, Gamma, and Delta variants of SARS-CoV-2, which were sometimes associated with new waves of infections throughout the world [6]. At present, Omicron is the leading and only disseminated variant. It is a highly divergent among the five variants of SARS-CoV-2, with a large number of mutations, mostly in the spike (S) protein. Computational and sequencing investigations initially divided the Omicron variant (B.1.1.529.1 or BA.1) into three sublineages, namely BA.1.1 (B.1.1.529.1), BA.2 (B.1.1.529.2), and BA. 3 (B.1.1.529.3) [7]. In the evolutionary lineage of the Omicron variant, the BA.1.1 sublineage evolved first, followed by the BA.2, and BA.3 [8]. BA.1.1 is the first dominant sublineage of Omicron and is believed to be responsible for the Omicron wave [9]. A recent study in South Africa uncovered two more sublineages, designated BA.4 (B.1.1.529.4) and BA.5 (B.1.1.529.5) that are associated with increased risk of re-infection in vaccinated individuals [10]. Moreover, BA.2.12.1 and BA.2.13 are additional developing sublineages. These evolved sublineages are spreading faster than other circulating strains, particularly BA.2, which could lead to another wave of COVID-19 cases due to immune evasion [5].

The S-protein of SARS-CoV-2, including Omicron, is a critical determinant of entry, transmissibility, and interaction points between the virus and the human immune system. Therefore, the S-protein is a prime antigen candidate for vaccine design. S-protein contains S1 and S2 subunits and furin protease cleavage sites [11,12]. The Omicron's S-proteins include the full-length trimer, with three receptor-binding S1 heads perched on top of a trimeric membrane fusion S2 stalk [13]. The S1 subunit contains an N-terminal domain (NTD) which is connected with a receptor binding domain (RBD) that specifically binds with angiotensin-converting enzyme 2 (ACE2). On the RBD, there is also a specific part that actually connects to ACE2 which is called the receptor-binding motif (RBM). On the S2 subunit, there is a specific part, the fusion protein (FP) whose function is to fuse and drill the host cell membrane to facilitate viral particles entering the cells. The S2 cannot perform its function until the S1 is separated. During viral infection, host target cell protease, TMPRSS2 cleaves the S1 and S2, and even within S2 as well [14]. Then, the S1 is dissociated and S2 is opened up and undergoes a dramatic structural change, such that it elongates, sort of twists and turns around and then it fuses with the cell membrane through the fusion protein which is necessary for the virus to enter into the target cells [14]. In the current variant, Omicron (BA.1) harbors more than 32 mutations in their S-protein, the highest number compared to other variants of SARS-CoV-2 [15,16]. Some of the mutations in the Omicron S-protein may be associated with the possibility of escaping the immune response. Concerns about escaping immunity from the vaccine have changed our understanding of the ongoing COVID-19 pandemic ending. The world was misled by the idea that global vaccination alone was sufficient to control the ongoing pandemic. Indeed, the variants of SARS-CoV-2 highlight the importance of variant-independent potential therapeutics with vaccination as well as public health preventive measures in controlling the ongoing pandemic. In this review, I discuss the current status of Omicron variant mutations and mechanisms of immune responses in the context of immune escape from vaccines. I also discuss the potential interventions that are independent of the variants of SARS-CoV-2.

## 2 Omicron variant mutations and their effects

The genetic sequence of SARS-CoV-2 contains about 29,881 base pairs (bps) encoding 9860 amino acids [17]. Although the virus has a proofreader that keeps it under control, the SARS-CoV-2 virus has mutated over time since the pandemic emerged. There are approximately 22,000 amino acid mutations and more than 13,000 insertions/deletions across the SARS-CoV-2 genome since the onset of the COVID-19 pandemic, which increases viral infectivity, worsens the disease, and reduces therapy or vaccine efficacy [[18], [19], [20]]. Most mutations were in open reading frame 1 ab (ORF1ab) (73%), followed by S-protein (13%) and nucleocapsid (4%) [19,21]. The Omicron variant harbors up to 50 mutations in its genome from its progenitor, the Wuhan type, and more than 32 in the S-protein, including three deletions and one insertion [15,16,22]. Interestingly, scientists have uncovered multiple impacts of the mutations in Omicron variant or other variants of SARS-CoV-2. In particular, the mutations in the Omicron variant and its sublineages can cause a variety of significant changes in the virus's properties, such as evasion of vaccine-induced immunity [[23], [24], [25], [26]], increasing the binding capacity of S-protein to ACE receptor [24,[27], [28], [29], [30]], effective proteolytic priming of S1and S2 with TMPRSS2, which significantly improves cell surface entry [27], and increase cellular invasion via the endocytic pathway [31]. However, despite all the consequences associated with the mutation, it is noteworthy that the disease severity caused by the Omicron variant is not significantly increased and the diseases remain mild or moderate [32].

Table 1 depicts S-protein mutations, their distribution, and their effects on the Omicron variant (BA.1) and other variants. There are 32 distinct mutations in the Omicron variant (B.1.1.529.1) S-protein. Of these, twenty-three mutations, including G339D, S371L, S373P, S375F, N440K, G446, S477 N, E484A, Q493R, G496S, Q498R, Y505H, A67V, Δ 143–145, Δ 211, Ins214EPE, T547K, N679K, N764K, D796Y, N856, Q954H, N969K, and L981F, were unique to the variant which had not been documented in any previous variants. The other nine mutations, namely K417 N, T478K, N501Y, Δ 69–70, T95I, G142D, D614G, H655Y, and P681H were found to overlap with previous variants of SARS-CoV-2, including Alpha, Beta, Gamma and Delta [33]. In the distribution of mutations in S-protein, half of them were in the RBD, such as G339D, S371L, S373P, S375F, N440K, G446, S477 N, T478K, E484A, Q493R, G493S, Y4958, Y4958, Y518, Y508, Y518 which are associated with increased transmissibility of the Omicron variant [34]. Specifically, the S477 N, Q498R, and N501Y mutations were found to be associated with increased binding of the Omicron S-protein to the ACE2 receptor, which may increase transmissibility and infectivity. In earlier variants such as Alpha, Beta, and Gamma, the presence of the critical mutation, N501Y, is associated with an increased binding capacity of the S-protein to the ACE2 receptor [[35], [36], [37]]. In addition, the presence of T478K and E484A mutations in the Omicron variant has been found to increase neutralizing antibody resistance and is associated with immune escape [7,37]. Similarly, the presence of such mutations along with H69/V70 deletion in earlier variants of SARS-CoV-2 has been associated with enhanced immune evasion [35].

**Table 1:** Illustration of S-protein mutations, their distribution, and their effect on Omicron variants (BA.1) and other variants.

Location of mutations		Mutations	Overlaps with other variants	Impacts of the mutations	Ref.
S1	RBD	G339D	–	Increases transmission, severity, and binding affinity of S-protein with ACE2.	[46]
		S371L	–	Increases transmission and resistance to specific antibodies.	[47]
		S373P	–	Increases infection rate and binding affinity of RBD with ACE2.	[46]
		S375F	–	Increases transmission, infection rate, and immune escape.	[48]
		K417 N	Beta	–	–
		N440K	–	Increases infection rate and binding affinity of RBD with ACE2.	[47]
		G446	–	Increases infection rate.	[49]
		S477 N	–	Increases the binding affinity of S-protein with ACE2, increases resistance to human convalescent plasma neutralization, and decreases neutralization to vaccine-induced sera.	[49]
		T478K	Delta	Increases transmission and infection rate and resistance to convalescent plasma.	[46]
		E484A	–	Enhances transmissibility.	[46]
		Q493R	–	Increases infection rate and contributes to immune escape.	[46]
		G496S	–	Increases infection rate and decreases protein stability.	[46]
		Q498R	–	Increases infection rate and decreases protein stability.	[46]
		N501Y	Alpha, Beta, and Gamma	Increases infection rate. Increases binding affinity to ACE2 & enhanced immune invasion.	[48]
		Y505H	–	Increases infection rate.	[48]
	NTD	A67V	–	–	–
		Δ 69–70	Alpha	S gene target failure and decreases antibody neutralization.	[50,51]
		T95I	Delta	Increases viral binding affinity, transmissibility, and immune escape possibility.	[50]
		G142D	Delta	–	–
		Δ 143–145	–	Increases viral binding affinity, transmissibility, and immune escape possibility.	[52]
		Δ 211	–	Unknown.	–
		Ins214EPE	–	Unknown.	–
	S1/S2	T547K	–	Stabilize the RBD.	[53]
		D614G	Alpha, Beta, Gamma, Delta	Increases infectiousness, transmissibility, and viral load.	[48,54]
		H655Y	Gamma	Increases transmissibility, infectivity, and resistance to monoclonal antibodies.	[51]
		N679K	–	Increases transmissibility and infectivity.	[48]
		P681H	Alpha	Increases transmissibility and infectivity.	[48]
S2 subunit		N764K	–	These changes may be associated with changes in the electrostatic potential of the S-protein, which may improve the transmissibility of Omicron variants. The immunogenic relevance of such loci is not yet known.	[51]
		D796Y	–		
		N856	–		
		Q954H	–		
		N969K	–		
		L981F	–		

Note: ‘-’ Represents mutations in the original Omicron variant (BA.1), Δ represents deletion, and ins represent insertion, S1 and S2 subunit of the S-protein, receptor binding domain (RBD), N-Terminal domain (NTD).

The Omicron variant has five mutations in the S1/S2 cleavage site, namely T547K, D614G, H655Y, N679K, and P681H (Table 1). Of these, the last three mutations occur in the furin cleavage site (S1/S2), which may enhance the fusion of virus and host cell membranes, thereby increasing transmissibility and infectivity. Delta spike has a P681R mutation at the furin cleavage site that has been shown to increase the cleavage of full-length S to S1 and S2 by human protease, TMPRSS2, thereby increasing transmissibility (Fig. 2A) [18,38,39]. However, Omicron appears to be more transmissible than Delta and has the P681H mutation in its furin cleavage site. Note that in Delta, proline was replaced by arginine, while proline was replaced by histidine in Omicron (Fig. 2). It is worth noting that the charge on arginine and histidine is the same. So, from a charge point of view, the furin cleavage site of Delta and Omicron, both are positively charged, meaning that the electromagnetic force and shape may not change much. This suggests that the Delta and Omicron furin cleavage sites may have a similar function, meaning that the behavior of TMPRSS2 may not be affected. However, two additional mutations in the Omicron S-protein near the furin cleavage site, namely H655Y + N679K, have been shown to accelerate S1/S2 cleavage by the protease and enhance the fusion of virus and host cell membranes, thereby increasing Omicron's ability to infect and replicate [40,41]. The Omicron S2 site contains six distinct mutations, including N764K, D796Y, N856K, Q954H, N969K, and L981F, which may be associated with viral entry and transmissibility into host cells (Table 1).

Table 2 depicts S-protein mutations and their effects in the Omicron sublineages. Over the past few months, the Omicron variant has appeared in multiple sublineages that differ from each other in the number of mutations and their level of infectivity [42,43]. BA.1.1 is the first sublineage with a specific mutation, such as R346K in the S-protein that causes immune evasion [29]. BA.2 has the new mutations, including T376A, L452, F486, and R408S that also confer immune evasion [32]. BA.2.12.1, and BA.2.13 has L452Q, and L452 M specific mutations, respectively, and all have a more considerable transmission advantage over BA.2 [33]. Both BA.4 and BA.5 have the L452R + F486V mutations [44]. The S-protein mutation at the L452R position is considered to be responsible for increased transmissibility. The Delta variant also had this mutation. The F486V mutation occurs in the S2 of the S-protein region near the attachment site in human cells. It is important to note that this mutation helps the virus dodge our immune system against vaccines. Therefore, these new sublineages of Omicron may be more infectious and able to evade the human immunity. Scientists hypothesized that these new sublineages of Omicron have more capacity to infect fully immunized people than the previous variants [33,44,45].

**Table 2:** Illustration of S-protein mutations and their effects in the Omicron sublineages [29,33,44].

Omicron variants	Additional mutations in the S-protein of the sublineage	Impact of the mutations
Original Omicron (BA.1)	–	–
BA.1.1	R346K	Increases transmission, severity, and immune evasion.
BA.2	T376A	Increases transmission, severity, and immune evasion.
	L452	Increases transmissibility.
	R408S	Unknown.
	F486	Unknown.
BA.2.12.1	L452Q	Have a larger transmission advantage than BA.2.
BA.2.13	L452 M	Have a larger transmission advantage than BA.2.
BA.3	R408S	Unknown.
BA.4, BA.5	L452R, F486V	Increases transmissibility and immune evasion.

Note: ‘-’ Represents mutations mentioned in Table 1 in the original Omicron variant (BA.1).

## 3 Epidemiology and severity of Omicron infection

There is strong epidemiological evidence that Omicron is highly infectious and spreads quickly, but causes milder disease than preceding variants of SARS-CoV-2 [55]. In particular, a South African study estimated that Omicron was 36.5% more transmissible than Delta [56]. Furthermore, studies from South Africa, the United Kingdom, and Denmark suggest that Omicron has a three to four-fold higher risk of infection than Delta and a reproduction number (R_0_) of 7 or greater, compared with 5 for Delta and 2.8 for the wild SARS-CoV-2 [57,58,59]. Despite having a high rate of COVID-19 cases during Omicron waves, the hospitalization rate was found to be lower (5%) than that of Delta waves (13.7%) [60]. Several preclinical studies have shown that Omicron has lower S-protein cleavage efficiency than its progenitor and Delta [31,61], leading to impaired syncytia formation, which may reduce the pathogenicity [22,62]. Efficient cleavage of the S-protein is important for SARS-CoV-2 virus entry into human cells which is mediated by TMPRSS2, and TMPRSS2-expressing cells are more abundant in the lungs than in the nose, throat, and airways [22]. It is important to note that SARS-CoV-2 has two different routes of entry to host cells; One is mediated by TMPRSS2 (TMPRSS2-dependent mechanism) and the second is mediated by cathepsin L cleavage at the S2 site (TMPRSS2-independent mechanism). TMPRSS2 facilitates viral entry to the plasma membrane since it is present in the cell membrane, whereas cathepsin L is in endosomes, which facilitates the endosomal entry route [63,64]. Previous variants play a potent role in transmission through a TMPRSS2 that induces strong syncytia formation in the lungs and exacerbates the damage from infection [65]. However, interestingly, the Omicron variant prefers the endosomal entry route (TMPRSS2-independent mechanism) to efficiently enter host cells over the plasma membrane entry route [31]. This observation was confirmed *in vitro* that Omicron replicates 10-fold less efficiently in the human lungs than Delta, resulting in less damage and less severe symptoms [31]. Omicron replicates rapidly in the upper respiratory tract, thus increasing the amount of virus released from the respiratory tract during inhalation [31,66], this may account for the higher transmissibility of Omicron but produces less severe disease than earlier variants [61].

Moreover, alteration in the electrostatic potential of the Omicron RBD and S1/S2 cleavage site was found to be associated with the binding capabilities of S-protein with the ACE2 receptor. A distinct trend of increasing positive electrostatic potential has been observed from the wild virus strain to Delta and the more recent Omicron variant [67]. Recently, it has been demonstrated that the significant increase in positive electrostatic potential at the RBD interface with ACE2 is the main reason for the increased affinity of ACE2 to the RBD in Omicron variants [67,68]. Since ACE2 has a negative electrostatic surface potential patch, it is reasonable to hypothesize that increasing the positive charge on the RBD of the S-protein would increase the viral contact affinity of the S-protein with the ACE2 receptor. Pawlowski et al. stated that the virus uses electrogenic modifications to change the electrostatic force between the RBD and ACE2, and the Coulomb attraction was found to be greater in Omicron than in the wild SARS-CoV-2 virus [68,69]. Therefore, it can be collectively stated that a high number of mutations in the Omicron variant results in a significant change in the electrostatic potential of the Omicron S-protein, which may be a plausible reason for its highly transmissible nature.

## 4 Mechanisms of immune responses elicited by the COVID-19 vaccine

A number of vaccines have been developed and administered for prophylactic and therapeutic purposes against SARS-CoV-2 infections. Four vaccines have been approved by the US Food and Drugs Administration (FDA) for emergency use including BNT162b2 manufactured by Pfizer-BioNTech, mRNA1273 by Moderna, and Ad26.COV2.S by Johnson & Johnson (J & J), and ChAdOx1 by AstraZeneca [70,71,72,73,74,75,76]. Other vaccines are also in use and many more are still in development. Most vaccines have been found to boost immunity and, more importantly, reduce COVID-19 infections, hospitalizations, and deaths in clinical trials [70,71,72,73].

**Fig. 1:**
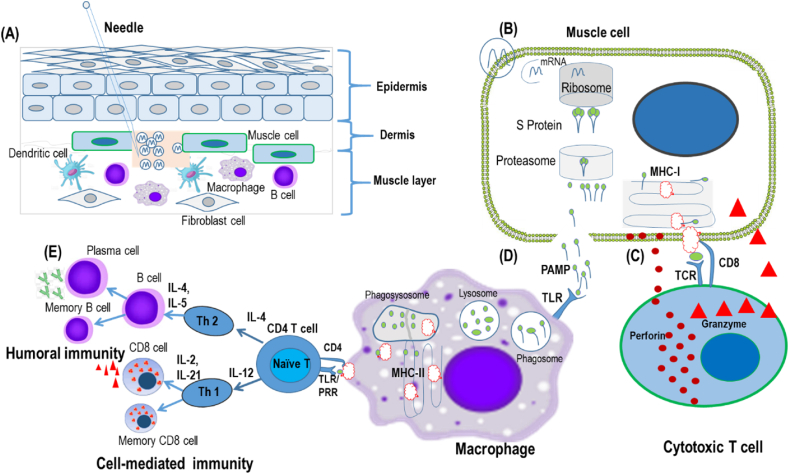
Mechanisms of immune responses elicited by the COVID-19 vaccines. Major steps involved in vaccine administration (A), translation, transcription, and presentation of S protein by MHC I molecules on the surface of muscle cells (B), followed by activation of cytotoxic T cells (C), and presentation of S protein by MHC II molecules on the surface of macrophages (D). Activated macrophages interact with naïve T cells and stimulate the production of humoral and cell-mediated immunity (E).

These vaccines use mRNA of S-protein as a template for the SARS-CoV-2 virus. Pfizer and Moderna both use lipid nanoparticles (LNPs) [77,78], and J & J and AstraZeneca used adenoviruses [75,79,80] as a potent and versatile delivery vehicle. Extracellular mRNA is readily digested by RNase in tissues, which is why naked RNA vaccines are not successful [81]. The adenovirus has double-stranded DNA that is transcribed into mRNA and finally translated into a protein. The active ingredient of all vaccines is mRNA. The J & J and AstraZeneca vaccines start one step earlier than Pfizer and Moderna. The end goal is the same, however, to make S-protein. Because the specific mechanisms of vaccine-induced immune responses against COVID-19 are currently incompletely understood, I have described general mechanisms of vaccine-induced immunity against viral infections, which may also apply to SARS-CoV-2 infection. A schematic diagram that gives an overview of the vaccine's immune response is presented in Fig. 1.

**Fig. 2:**
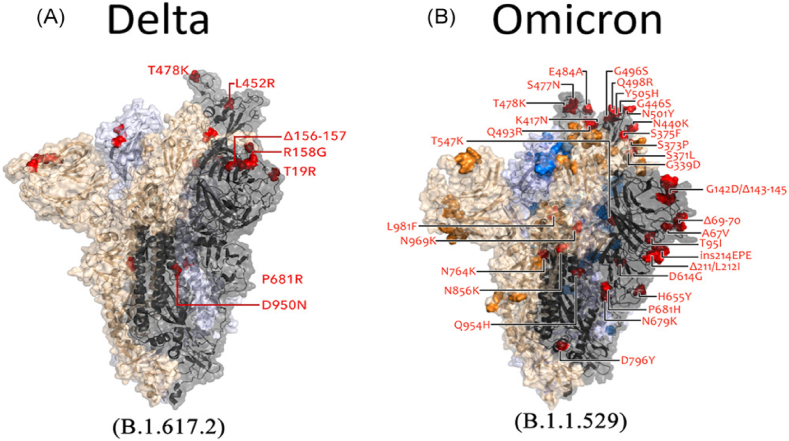
The three-dimensional (3D) structure of spike protein with mutations. A comparison of (A) Delta (B.1.617.2) and (B) Omicron variant (B.1.1.529) S-protein mutation (Image source: Journal of Medical Virology [101].

COVID-19 vaccines are administered intramuscularly into deltoid muscles for minimal adverse effects and most immunogenic responses [82,83,84]. The deltoid muscle contains many cells, such as muscle cells, fibroblasts, immune cells, (mostly dendritic cells, DCs, and macrophages), and natural killer (NK) cells (Fig. 1A). In the J & J and AstraZeneca vaccines, the adenovirus binds to the surface of the host cell and, subsequently, is internalized into an endosome that cleaves the viral shell and releases the DNA. This DNA enters the nucleus and is transcribed into mRNA. This mRNA then leaves the nucleus to the cytoplasm. As with adenovirus-based vaccines, the LNPs wrapped mRNA binds to the cell surface and is internalized, subsequently breaking down the LNPs shell, and releasing the mRNA into the cytoplasm (Fig. 1B). Free mRNA in the cytoplasm binds to the ribosome, a small machine that picks up genetic materials and synthesizes the protein that is encoded in the genetic materials. A mammalian cell can contain about 10 million ribosomes and spends up to 60% of its energy making one protein, and 80 different types of proteins are synthesized [85]. In the case of COVID-19 vaccines, it will produce the S-protein. After synthesizing S-proteins, cells do not let them out, like the hormone-secreting cells. Inside the cell, S-proteins are fed to another tiny cellular machine called the proteasome, which breaks them down into smaller pieces. The small pieces of S-proteins are loaded on special proteins which are called major histocompatibility complex. There are two types of MHC, such as MHC I and MHC II. Although MHC I is expressed by all cells, expression of MHC II is restricted mainly to DCs and macrophages [86,87,88]. These cells take up a foreign antigen and present it on their surface and then interact with other immune cells, referred to as ‘professional’ antigen-presenting cells (APCs). The small pieces of S-proteins are transported to the endoplasmic reticulum (ER) through a channel called transporter-associated antigen processing (TAP) where they are loaded onto nascent MHC I. The MHC I loaded S-protein goes to the cell membrane and is presented on the cell surface in a controlled manner (Fig. 1B). Once they are presented on the cell surface, cytotoxic T (CD8^+^) cells recognize and bind to MHC I- complex. Upon binding to the MHC I complex via T cell receptor (TCR) and CD8 protein, the T cell become activated (Fig. 1C). In the presence of IL2, CD8^+^ cells secrete perforins and granzymes, causing muscle cell apoptosis and breakdown. When the cell breaks down, S-proteins and some mRNAs are released from the cells. S-proteins and mRNA are immediately phagocytized by APCs, specifically, macrophages and DCs. After phagocytosis, macrophages form a vesicle around the S-protein called a phagosome. The phagosome is associated with the lysosome which is filled with acid bleach that breaks down S-protein. Once again broken down, the S-proteins are loaded in the ER which forms the MHC II. The MHC II-S protein complex comes out from the ER and goes into the phagolysosome where S-proteins are broken down into small pieces. The MHC II and S protein complexes move to the cell surface and are displayed in a regulated fashion (Fig. 1D). Now the complex in immune cells (macrophage) connects with naïve T cells or T helper (CD4^+^) cells to induce activation. The activated naïve T cells convert into either the T helper 1 (Th1) cell or the Th2 cell (Fig. 1E). However, the decision-makers of the Th1 and Th2 pathways are still unknown. There are several hypotheses exist to postulate the pathways. The PAMP and cytokine milieu hypotheses include what type of receptor binds to the pathogen/antigen to determine what type of immune response will occur. Given that activation of innate immune cells, particularly macrophages and DCs, occurs through pathogen-associated molecular patterns (PAMPs). The PAMP is recognized directly by the host pattern recognition receptors (PRRs) and toll-like receptors (TLRs). If TLRs bind to pathogens, macrophages/DCs will attempt to trigger the Th1 pathway. On the other hand, if PRRs are engaged in binding to pathogens, the Th2 pathway may be triggered [89,90]. Furthermore, decision-making is also influenced by tissue factors. Of course, when a pathogen is present, the tissue will begin to be damaged and local inflammation will begin, and local cytokines, chemokines are released by the inflammatory process, such as but not limited to ILs, TNFs, and heat shock proteins. The naïve T cells do not know whether to be Th1 or Th2 pathway. When macrophage is connected with the naïve T cells, there is to be costimulation depending upon the type of T cell receptors (TCR and PRR) or cytokines and chemokines available. These connecting cells, macrophages, or DCs will decide which cytokines will then influence the pathway [90]. For example, if IL12 is released into the environment at that time, the naïve T cell will go into the Th1 pathway and if IL4 is released, the naïve T cell will go into the Th2 pathway. There is another theory called the threshold hypothesis. This is important because the pathway is selected by the vaccine dose or a load of pathogens present in this interaction of macrophages with naïve T cells. It also determines why a person's immune response is humoral versus cell-mediated immunity [90,91]. This hypothesis said, if the antigen is less amount, then usually there is no response. If the antigen amount or pathogen load is intermediate, then the Th2 pathway is taken which is a humoral immunity pathway. If the pathogen/antigen load is too high, then the Th1 pathway is selected which is a cell-mediated immunity pathway. It could really depend upon the dose of the vaccine/pathogen to decide what kind of immune response will become active.

In presence of IL 4 and IL 5, the Th2 cell activates B cells to convert plasma cells which produce and secrete antigen-specific antibodies. The antibodies travel everywhere around through blood and neutralize the specific pathogen and thus provide a humoral immune response. And in presence of IL2 and IL21, the Th1 cell activates cytotoxic T cells to secrete perforins and granzymes that directly kill the infected cells, and thus provide the cell-mediated immune response (Fig. 1E). Both of the pathways also produce specialized long-lived cells called memory B and T cells for future protection.

Reduced humoral immune response against the Omicron variant has been reported in studies using plasma specimens from individuals with two doses of COVID-19 mRNA vaccines [92], and patients with previous SARS-CoV-2 infection [93]. This does not mean that it also suppresses T cell-mediated immunity. These vaccines have been reported to elicit robust T cell-mediated immune response that contributes to significant protection against hospitalization or death [60,94,95,96] and is likely, it plays a central role in the control of SARS-CoV-2 infection, including omicron, but their importance has been relatively underestimated until now. Similarly, innate immunity has a rapid and breadth-spectrum of activity that also plays an important role in terms of preventing morbidity, hospitalization, and even death of patients. Vaccines can be looked at on many endpoints, they can prevent infections, transmission, hospitalizations, and death to a certain degree, of course, and nothing is 100%.

## 5 Can Omicron escape immunity generated by vaccines or previous infection?

With the emergence of new variants of SARS-CoV-2 due to the mutation, antibodies generated by existing vaccines may lose the ability to neutralize different variants (148,149). A key question now is, to what extent do mutations in omicron variants evade immunity against vaccines and/or previous infections? The previous evidence for the immune escape, the genetic sequence of SARS-CoV-2 was different at about 50% and 79% from its cousins, Middle East respiratory syndrome coronavirus (MERS-CoV) and SARS-CoV, respectively [97]. A 50 bps change in Omicron means 0.167% of the genetic sequence is different from their ancestor, a percentage change too small to really evade immunity. A different line of evidence is that measles and polio viruses have high mutation rates, showing that 95% of people still have antibodies that neutralize these viruses very well with vaccination [98,99]. The measles vaccine has been around for 70 years, and poliovirus vaccine for a decade.

As mentioned above, the S-protein, the mediator of the host cell entry and the main target of neutralizing antibodies, has been used as the sole immunogen to produce vaccines [15,16,22] and Omicron's S-protein contains 32 mutations compared to wild‐type, half of them is in RBD (Fig. 2B and Table 1). Given that most mutations have little or no effect on virus behavior. However, depending on where mutations are located in the virus genome, they can affect virus behavior, such as infectivity and virulence. The entire sequence of the wild-type SARS-CoV-2 S-protein contains 1273 amino acids, 1271 in the Delta, and 1270 in the Omicron, and is encoded by 3831 bps [100,101]. A part of the S-protein exposed to an antibody to which the antibody binds is called an epitope. The 1270 amino acids make a protein that is three-dimensional (3D) in structure, S-protein in Omicron and has various epitopes on it (Fig. 2). From this 3D structure of the S-protein, we can imagine that not every single epitope of this protein will be available for antibody binding. It is reasonable to assume that some epitopes will fold and move inside so fewer numbers will be exposed outside for antibody binding. In general, about six to nine amino acids are combined to form an epitope that can be recognized and bound by an antibody. Given that the Omicron's S-protein has 1270 amino acids if we divide them by 6 that would make 211 epitopes linearly. It is known that epitopes, especially on B cells and T cells, are linear and conformational, but their exact proportions are not yet known [102]. When a virus or a vaccine enters our body, the immune system produces different types of antibodies that bind to various epitopes. The antibodies can be classified into class 1, class 2, and class 3, targeting a slightly different site of the S-protein and then neutralizing the SARS-CoV-2. For example, a mutation called E484K changes the shape of the site that class 2 antibodies identify, making them less powerful. Omicron harbors E484A mutation in this site and similar changes in the sites for the other two classes of antibodies (Table 1). Thirty two (32) bps of the Omicron S-protein has changed, not every single bp change will fail every single relevant antibody. In fact, some changes are silent, even electrochemical, electromagnetic, phenotypic, or shape changes, nothing happens and remains the same. The point is that we have 32 bps change between 200/300 epitopes, these 32 bps changes can make about 5–8 epitopes. Some of them will overlap. But in large 3D images, many epitopes will be found. Furthermore, if someone had a previous infection, they would not only have epitopes for the S-protein, but they would also have antibodies against other surface proteins, including membrane (M), envelope (E), nucleocapsid (N) proteins, and even non-functional proteins. There are much more epitopes available for their antibodies to have attempted to bind with. Therefore, if the virus has some mutations that cannot escape immunity, it will escape a small part of the immune system.

Table 3 shows that vaccine efficacy/effectiveness that was lower for the Omicron variant than for the Delta at all-time intervals after primary courses and booster vaccination. Specifically, 14–28 days after the booster dose, mRNA vaccine efficacy against Omicron drops from 93% to 74%, but the vaccine can still reduce the risk of hospitalization and death from Omicron-associated COVID-19 [103,104,105]. In a recent study, Nielsen et al. [106] compiled data from Danish nationwide resources and determined the vaccine effectiveness (VE) of the primary COVID-19 vaccination series against SARS-CoV-2 re-infection, COVID-19 hospitalization, and mortality. This study showed that vaccines are still effective against SARS-CoV-2 re-infection during periods with SARS-CoV-2 variants Alpha, Delta, and Omicron, ranging from 71%, 94%, and 60%–62%, respectively, and lasting up to 9 months. These results are consistent with a Qatar study that showed a 55.1% VE against re-infection with Omicron after two doses of a COVID-19 mRNA vaccine and 77.3% after three doses [107,108]. Furthermore, several studies looking at humoral immunity against Omicron showed that the increased levels of neutralizing antibodies owing to booster dose or a combination of natural infections so that antibody neutralization against Omicron in these groups is more or less equivalent to the neutralization seen against Delta variant in those who are booster vaccinated [53,109,110,111,112,113]. Moreover, recently, BNT162b2 and mRNA-1273 were shown to neutralize Omicron and previous variants with significantly increased cross-reactive broad-neutralizing antibodies 9–12 days after the second dose [114]. Moderate levels of neutralizing antibodies can protect individuals from serious diseases, hence researchers always try to extrapolate levels of antibodies to determine vaccine effectiveness. However, antibody levels were quite variable among hospitalized COVID-19 patients [115]. We know that the vast majority of patients can successfully fight off COVID-19 infections, even with very low antibody levels, and of course, this point indicates other aspects of the immune system, particularly T cell-mediated immunity. BioNTech and Pfizer claimed that two doses of the vaccine should still protect against severe disease because the vast majority of surface structures of the Omicron S-protein targeted by T cells, which usually appear after vaccination, are not affected by mutations in the Omicron [116]. A recent bioinformatics-based study looking at Omicron's T cell epitopes showed that most of the predicted Omicron S-protein T cell epitopes are not mutated in this variant, suggesting that Omicron's existing T cell immunity from vaccination or natural infection is not affected [117]. Moreover, several recent studies have shown that T cell immunity still confers protection against Omicron variant elicited by vaccination or natural infection [114,117,118,119,120]. A recent *meta-analysis* of data from 14,826,0342 participants in 13 studies found that the COVID-19 vaccine effectively reduces infections from the Omicron variant [121]. Most strikingly, the unvaccinated have a five-eight-fold higher chance of being admitted to the hospital and five-time more likely to get re-infection with Omicron compared to those who are primarily vaccinated [95,122], it is noteworthy that pre-existing immunity, including humoral, cell-mediated, and innate immunity, caused by vaccines or previous infections, is still strongly defensive against Omicron-related COVID-19. Of course, we can't say this 100%, but it seems remarkably protective.

**Table 3:** The efficacy of FDA approved vaccine against symptomatic disease caused by SARS-CoV-2 variants [103,104].

Vaccines	Effectiveness (95% Cl) against symptomatic disease				
**mRNA-1273**	Alpha	Beta	Gamma	Delta	Omicron
14 days after dose 1	82 (80, 84)	–	89 (76, 95)	70 (64, 76)	48 (43, 53)
7 days after dose 2	92 (88, 95)	–	–	95 (91, 97)	75 (71, 78)
**Booster dose 1**					
7 days after the booster	–	–	–	93.7 (93, 94)	74 (73–75)
14–28 days after the booster	–	–	–	96.6 (96,97)	74 (73–75)
**BNT162b2**					
14 days after dose 1	67 (65, 68)	50 (15, 70)	63 (54, 70)	57 (53, 61)	43 (40, 45)
7 days after dose 2	89 (87, 90)	87 (8, 98)	88 (73, 94)	92 (90, 94)	65.5 (64, 67)
Booster dose 1					
7 days after booster 1	–	–	–	92 (92, 93)	67 (66, 67)
14–28 days after the booster	–	–	–	95 (95, 96)	68 (66.7, 68)
**ChAdOx1**					
14 days after dose 1	63 (59, 66)	84 (−13, 98)	41 (12, 60)	68 (57, 76)	–
7 days after dose 2	91 (62, 98)	–	–	87 (69, 95)	59 (40, 57)
**Booster dose 1**					
7 days after the booster	–	–	–	77 (55, 88)	58 (38, 71)
14–28 days after the booster	–	–	–	82 (71, 89)	56 (44, 65)

## 6 Variant independent therapies

Although vaccines have a huge role to play in preventing the spread of COVID-19 and reducing hospitalizations and mortality, patients with immunocompromised and underlying comorbidities cannot be protected by vaccination alone. Moreover, vaccines have some degree of variant-dependent activity, indicating new challenges in preventing and controlling Omicron and/or emerging new variant-associated COVID-19 [123]. Therefore, along with vaccines, it is essential to identify potential therapeutics targeting the pathophysiology that acts independently of the genetics of SARS-CoV-2 and its variants. In the following, I have listed several drugs that have potent antiviral, antioxidant, and anti-cytokine activity.

### 6.1 Remdesivir

**Fig. 3:**
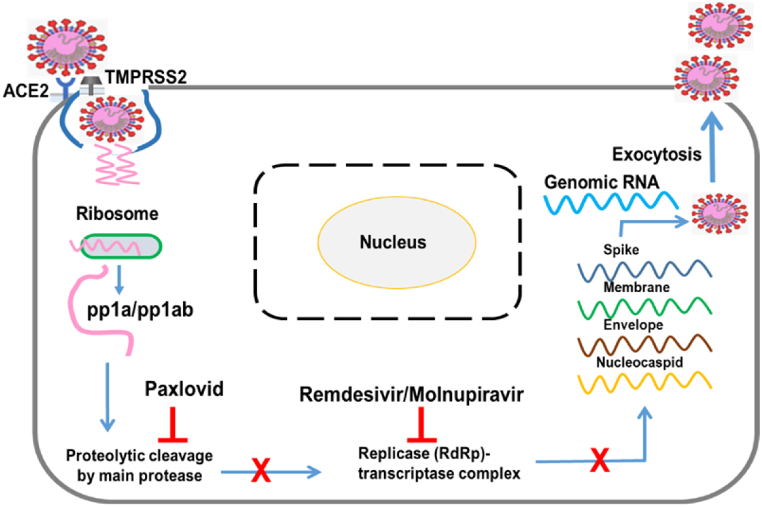
**The mechanism of SARS-CoV-2 viral replication and the main targets of antiviral drugs (Paxlovid**™**, Remdesivir, and Molnupiravir).** The attachment of SARS-CoV-2 to the host cell is mediated by binding the viral S protein to the ACE2 receptor. Following the proteolytic cleavage of the S1 unit by a membrane serine protease, TMPRSS2, a fusion of viral and host cell membranes is initiated by the exposed S2 unit. Alternatively, Omicron may invade host cells during endosomal uptake. The released RNA is translated by the ribosome of the host cell. Polyprotein pp1a/pp1ab is cleaved by viral main protease (M^pro^). The released structural and non-structural proteins form the replicase (RdRp)–transcriptase complex, which initiates the machinery of viral RNA synthesis. Viral structure proteins and genomic RNA form new viruses, which are released by exocytosis. SARS-CoV-2 replication can be inhibited at different stages of the virus's life cycle: viral major protease inhibition by Paxlovid™, RNA replication (RdRp) inhibition by Remdesivir/Molnupiravir.

Remdesivir is a broad-spectrum antiviral drug that acts against various RNA viruses, such as *Coronaviridae*, *Paramyxoviridae*, and *Filoviridae* [124,125,126,127,128]. It was originally developed to combat infections from Ebola and related viruses [124]. Remdesivir is a pro-drug of the adenosine analog, which is converted to active metabolites, GS-44152, nucleoside triphosphate (NTP), by the host [129]. The NTP competes with adenosine triphosphate (ATP) for incorporation into the nascent RNA strand [130] (Fig. 3). As a nucleoside analog, remdesivir shuts down viral replication by acting as a ‘false building block’ that is dysfunctional and thus terminates replication/transcription (Fig. 3). In *an in vitro* study, Wang et al. [131] revealed that remdesivir is highly effective against SARS-CoV-2 infections in human respiratory epithelial cells. Based on existing data from *in vitro* studies [131], multi-site several randomized clinical trials (RCTs) of remdesivir among hospitalized adults COVID-19 patients have been completed [132,133]. RCTs are the gold standard for the assessment of potential therapeutics efficacy. An RCT of remdesivir showed that both a 10-day course and a 5-day course shortened the recovery time in patients hospitalized with COVID-19 [133]. Another double-blind, RCT of a 3-day course of remdesivir had an acceptable safety profile and resulted in an 87% lower risk of hospitalization or death than a placebo within 7 days of the appearance of symptoms [134]. Based on the data from the RCTs, Remdesivir (Veklur^R^) has been approved by the US FDA for the first time as a first antiviral drug for the treatment of mild to moderate COVID-19 symptoms patients and are a high-risk population for progression to severe COVID-19, including hospitalization or death who are 12 years of age or older [135]. But the drug has to be intravenously medicated, which drastically limits its utility, especially in areas where infusion clinics may not be readily available. Moreover, the solidarity trial conducted by the World Health Organization (WHO) did not find such a benefit [136]. These emphasize the need to look for new antiviral drugs.

### 6.2 Molnupiravir

Molnupiravir is an antiviral drug that recently has been proven in randomized-placebo controlled clinical trial for the treatment of COVID-19 patients [137]). Molnupiravir is a nucleoside analog that increases the frequency of viral RNA mutation by RNA-dependent RNA-polymerase (RdRp) and it inhibits various viral replication including SARS-CoV-2 in animal models and humans [138,139,140,141,142,143]. When the Molnupiravir enters the cell, it is converted to its active form, β-d-N^4^-hydroxycytidine triphosphate (M). The M can be used by the SARS-CoV-2 viral RdRp, as a substrate instead of cytidine triphosphate or uridine triphosphate. The RdRp is an extremely important enzyme for SARS-CoV-2 virus replication. The M-containing RNA can be used as a template then leads to mutated RNA products, which do not support replication of intact new viruses (Fig. 3) [140], as predicted by the ‘error catastrophe’ model [138,144,145]. Since the mechanism of action of Molnupiravir is independent of mutations in the spike protein, it is expected to work against Omicron. Indeed, Molnupiravir had similar efficacy against the Alpha, Beta, Gamma, Delta, and Omicron *in vitro* study of VeroE6-GFP cells though there are no clinical studies yet to indicate the effectiveness of Molnupiravir in people infected with Omicron [146]. A double-blind RCT was conducted in non-hospitalized adults with 800 mg of oral covid-19 medication daily for a 5-day course before the Omicron wave (May 2021 to November 2021). In the analysis of1433 participants, the percentage of participants who were hospitalized or died through day 29 was significantly lower: 6.8% (48 of 709) in the intervention group as compared with 9.7% (68 of 699) in the placebo group (RR, −3.1%; 95% CI, −5.9 to −0.1) [137] (Table 4). In another study, Molnupiravir has been found to reduce the risk of hospitalization or death by 50% in non-hospitalized adult patients [147]. Based on the data, Molnupiravir has been authorized by the FDA for emergency use for the treatment of mild to moderate COVID-19 in the adult with positive results of direct SARS-CoV-2 testing who are in high risk for progression to severe COVID-19 including hospitalizations and death.

**Table 4:** Recent randomized clinical trials evaluating the therapeutic efficacy of several recent variant-independent drugs in COVID-19 outcome.

Drugs	Size	Dose	Outcome	Effects
Paxlovid™ [151,152]	1433	300 mg nirmatrelvir and 100 mg ritonavir twice daily for 5 days (orally)	Hospitalizations or death reduction by 89%	Mortality was significantly decreased (0/607 for Paxlovid™ group vs. 10/612 for placebo group); p < 0.0001
Remdesivir [134]	562	200 mg on day 1 followed by 100 mg for 3 days (IV)	Hospitalizations or death reduction by 87%	HR = 0.13; (95% CI, 0.03–0.59); P = 0.008
Monlupiravir [137]	1433	800 mg twice daily for 5 days (orally)	Hospitalizations or death reduction by 31%	HR = 0.69; (95% CI, 0.48–1.01)
Fluvoxamine [169]	1480	100 mg twice daily for 10 days (orally)	Hospitalizations or death reduction by 29%	Mortality was significantly decreased (1/548 for fluvoxamine vs. 12/619 for the placebo group). RR = 0·34, 95% BCI, 0·21–0·54.
Tempol [184]	248	800 mg daily for 14 days	No information yet	No information yet
Vitamin D [205]	76	0.532 mg on the day of admission and 0.266 mg on day 3, 7 and weekly	Significantly reduce the need for ICU (e.g. reduced the severity)	Mortality was significantly decreased (0/50 for vitamin D vs. 2/26 for placebo group)

### 6.3 Paxlovid™

Paxlovid™ is a combination product containing nirmatrelvir **[PF-07321332]** and ritonavir. The nirmatrelvir is a SARS-CoV-2 main protease inhibitor (M^pro^, 3CL^pro,^ or nsp5 protease inhibitor), a key enzyme that viruses need to multiply in the human body [148]. Inhibition of SARS-CoV-2 M^pro^ renders the virus incapable of processing polyprotein precursors, thus preventing viral replication (Fig. 3). Briefly, when the virus attaches to the cells through ACE2 receptors, it releases its viral RNA into the cell cytoplasm. Ribosomes in the RNA produce polyprotein chains. The long protein chains are cleaved by enzymes called proteases into smaller viral proteins. The viral protein forms a complex which then leads to RNA replication, finally, assembly and release of a new virus occur to complete the process. Since nirmatrelvir contained in Paxlovid™ blocks the main protease, 3CL, and prevents the breakdown of the polyprotein into smaller segments. This ultimately prevents RNA replication and stops further viral production. The Ritonavir is a CYP3A inhibitor included to increase nirmatrelvir plasma concentrations. Higher doses of ritonavir used to be used as an HIV protease inhibitor [149]. A recent *in vitro* study suggests that M^pro^ mutants of SARS‐CoV‐2 variants remain susceptible to nirmatrelvir. They identified the most prevalent M^pro^ variants including G15S, T21I, L89F, K90R, P132H, and L205V in different SARS-CoV-2 lineages including Beta, Lambda, and Omicron, and biochemical assay showed that nirmatrelvir has promising potency against all the variants, indicating that Paxlovid™ may be effective against the Omicron variant [150]. In a double‐blind, placebo‐controlled phase II/III trial conducted by Pfizer Inc. Paxlovid™ has been found to reduce the hospitalization and death risk by 89% when administered within 3 days of symptom onset (Table 4). Notably, the death rate was found to reduce with high statistical significance (p < 0.0001) [151,152]. A recent meta-analysis of 18,568 patient data from 41 RCTs comparing the efficacy of antiviral drugs in patients with COVID-19 found that malnupiravir and nimatrevir-ritonavir (Paxovid™) significantly reduced the risk of hospitalization and death. Paxlovid™ is more effective than malnupiravir in reducing the risk of hospitalization [153]. These findings raise new hopes that Paxlovid™ is a promising therapy for COVID-19 patients. Moreover, several clinical trials of Paxlovid™ are currently underway and will be disclosed soon [154,155,156,157]. Based on the existing potential efficacy against COVID-19, the FDA has given emergency use authorization to Pfizer's antiviral drug, Paxlovid™ in December of 2021 for the treatment of mild to moderate COVID-19 in adults and pediatric patients (12 years of age and older). It is designed to give early after diagnosis within 5 days of symptoms who are at high risk for progression to severe COVID-19, including hospitalization or death. Nirmatrelvir has been observed to be safe and well‐tolerated with no side effects at oral dosing up to 600 mg/kg daily in monkeys and 1000 mg/kg daily in rats for 14 days [158]. Paxlovid™ is the first oral antiviral drug at-home treatment for COVID-19 and is considered a ‘game changer’ drug as it does not require an intravenous infusion like remdesivir.

### 6.4 Fluvoxamine

**Fig. 4:**
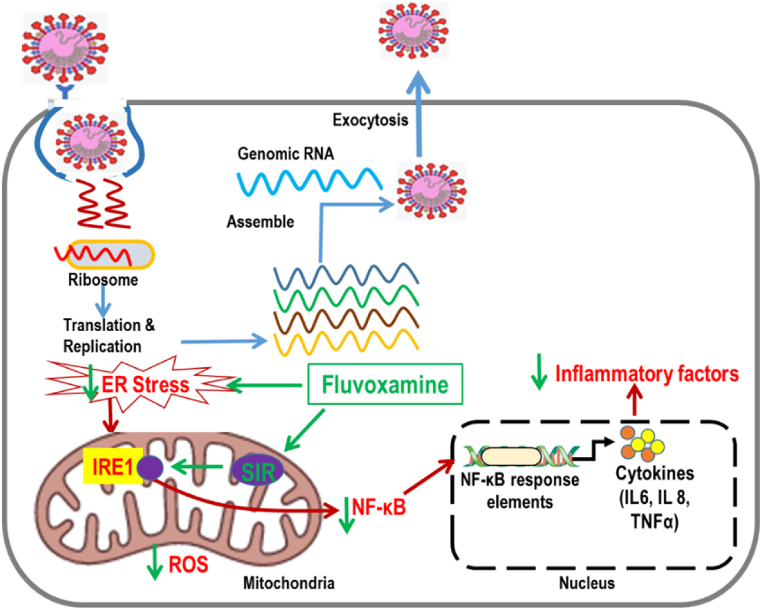
Proposed mechanism of fluvoxamine in the treatment of Omicron-associated COVID-19 patients. Fluvoxamine stimulates the Sigma1 receptor (SIR), a mitochondrial membrane that actually binds to IRE1 and essentially cools it down and inhibits it from performing many activities such as NF-κB stimulation that reduce cytokine storm. Moreover, fluvoxamine has a potent antioxidant activity.

Fluvoxamine is an FDA-approved drug for certain psychiatric conditions, such as major depressive disorder, seasonal affective disorder, obsessive-compulsive disorder, and post-traumatic stress disorder but there also seems to be a part that it plays in reducing oxidative stress and inflammation and even cytokine storm that is implicated at least in part some the hospitalizations related to COVID-19. The major function of fluvoxamine is known as a selective serotonin reuptake inhibitor (SSRI) that effectively increases the concentration of serotonin in the synaptic cleft or the area, causing an increase in stimulation of these serotonin receptors and finally, causing an excitatory pattern at the post-synaptic receptor and finally reliefs the above disorders [159]. The mechanism of action of fluvoxamine in COVID-19 is still unknown, but it has potent antioxidant and anti-cytokine activities that would be beneficial for improving COVID-19 outcomes (Fig. 4). Accumulation evidence suggest that the SARS-CoV-2 virus causes oxidative stress in the cells. Moreover, COVID-19 patients with obesity, diabetes, heart disease, aging, and immunosuppressive conditions exhibit elevated levels of reactive oxygen species (ROS) and oxidative stress [160]. The inositol reuptake enzyme one alpha (IRE1), a mitochondrial membrane protein can sense the increased levels of ROS in the cell, and through a number of complex processes, essentially, the IRE1 activates nuclear factor kappa beta (NF-κB) which enter into the nucleus and cause transcription of DNA to make inflammatory factors including cytokines, as in cytokine storm [161,162]. Oxidative stress coupled with the cytokine storm is a known hallmark of severe COVID-19 [160]. Besides being an SSRI, fluvoxamine stimulates the sigma 1 receptor (SIR), a mitochondrial membrane that actually combined with IRE1 and essentially cools it down and prevents it from doing a lot of its activity like stimulating NF-κB that causes a reduction in a cytokine storm [161,163]. Other suggested mechanisms possibly contributing to the prevention of COVID-19 include reduction in platelet aggregation, inhibition of histamine release from mast cells, and interference with endolysosomal viral trafficking [164,165].

For having such promising functions, scientists designed an RCT to see whether or not fluvoxamine would improve outcomes in COVID-19 and the result was published in November 2020 [166]. In this RCT (n = 152), fluvoxamine has been found to prevent clinical deterioration in adult outpatients infected with SARS-CoV-2. Subsequently, similar results were found in a prospective, non-randomized observational cohort study and meta-analysis of fluvoxamine in outpatients (n = 113) infected with SARS-CoV-2 [167,168]. Since the sample size was small, these studies strongly encouraged further double-blind RCTs using a large sample size. Subsequently, a large sample size (n = 1480) double-blind RCT found that fluvoxamine treatment at a dose of 100 mg for 10 days significantly reduced hospitalization or death by 29% (95% CI 0.54–0.93) in high-risk outpatients compared to the placebo group (Table 4) [169]. They also compared three compounds, fluvoxamine, metformin, and ivermectin. Metformin and ivermectin did not show beneficial effects. Apparently, fluvoxamine may be the most attractive drug candidate for early-stage of COVID-19. It has safety profiles, widespread availability, and is very cheap administered orally and also can be used for children and adolescents [170]. A recent meta-analysis of 4842 participants in eight studies combining Fluvoxamine with Paxlovid™ or Malnupiravir showed that these novel drugs were significantly effective in reducing mortality and hospitalization rates in Omicron-associated COVID-19 patients [171].

### 6.5 Tempol

Recently, the National Institute of Health (NIH) has highlighted Tempol as a potential therapeutic candidate for COVID-19 treatment. In fact, the hyper-inflammatory immune response attributed to a cytokine storm together with oxidative stress contributes to COVID-19 pathogenesis which is characterized by endothelial cell dysfunction and enodtheliitis that results in thrombosis which can damage the lungs and other vital organs that leads to heart attack and brain stroke and finally may cause death [160]. Tempol is claimed to possess “Three in one” activity, such as anti-inflammatory, antiviral and antioxidant, having such activity raised new hopes that Tempol would be a promising therapy for COVID-19 patients. Based on the activities, Adamis Pharmaceuticals has submitted an application seeking fast-track designation from the FDA for Tempol as a potential drug for COVID-19 management at home.

Tempol is a redox-cycling nitroxide that promotes the metabolism of ROS and improves nitric oxide (NO) bioavailability [172,173]. However, the decreased bioavailability of NO leads to endothelial cell dysfunction [174], which contributes to the release of coagulation factors which leads to thrombosis [160,175]. Tempol has superoxide dismutase (SOD) mimetic activity that catalyzes the dismutation of superoxide (O_2_^−^) into O2 and H_2_O_2_. Preclinical studies of Tempol have demonstrated decreased ischemia/reperfusion-induced diseases by its free radical scavenging activity [176,177,178]. Moreover, Tempol has been found to decrease oxidative stress by activating the nuclear factor erythroid 2-related factor 2 (Nrf2) pathway. Tempol has been shown to decrease pro-inflammatory cytokines, even cytokine storm via inactivation of the NF-κB pathway [179]. Preclinical studies of Tempol have demonstrated decreased inflammatory cytokines from a variety of cell types [172,179]. Recently, the anti-cytokine effects of Tempol have been investigated on COVID-19 patients. The authors found a significant decrease in multiple T cell and APC-derived cytokines from the cells of COVID-19 *in vitro* [180]. Moreover, Tempol has been found to decrease COVID-19 infections by inhibiting the RdRp and blocking the SARS-CoV-2 viral replication *in vitro* [181]. The previous report showed that Tempol directly reacts with the iron-sulfur (Fe–S) cluster and it was able to oxidize and disassemble the Fe–S cluster [182]. In fact, the Fe–S cluster present in the catalytic subunit of the RdRp is essential for SARS-CoV-2 replication and spread. In a recent report, Maio et al. showed that Tempol and remdesivir synergistically inhibited the activity of the RdRp and blocked SARS-CoV-2 replication [181].

Overall, although most of the studies are preclinical using animal models and cell cultures, it is thought that Tempol can suppress cytokines production and protect organs by calming inflammation, decreasing oxidative stress, and the clumping of platelets [172,179,180,181,183]. Several RCTs are urgently needed to clarify the role of Tempol in reducing SARS-CoV-2 infection. A Phase 2/3, adaptive, RCT was started to examine the effects of Tempol in preventing COVID-19 hospitalization in patients with SARS-CoV-2 infection (Table 4) [184]. Although the results of this study will be accessible in near future and will provide valuable information on to what extent Tempol benefits COVID-19 patients, the strong antiviral, anti-cytokine, and antioxidant activities may be a potentially beneficial strategy in early and severe SARS-CoV-2 infection.

### 6.6 Vitamin D

Vitamin D is a fat-soluble that is endogenously synthesized in our body through sunlight exposure and also can be obtained by supplementation. It is known that serum levels of 30 ng/mL of vitamin D are essential for boosting immunity [185], while below 20 ng/mL is considered vitamin D deficiency, and levels between 21 and 29 ng/mL are considered insufficient [186]. Overall, the production of disease in an individual, including COVID-19, depends largely on host immunity, such as innate and adaptive immunity, so boosting immunity is a very important strategy to fight disease. Vitamin D boosts the innate immune system which reduces the viral load and reduces the over-activity of the adaptive immune system and cytokine storm, thereby reducing COVID-19 mortality [187]. There is ample evidence that vitamin D is a potent therapeutic agent against SARS-CoV-2 infections (Table 4) [187,188,189,190]. Furthermore, the combined supplement of vitamin D and l-cysteine, COVID-19, has been shown to be more effective in reducing the risk of oxidative stress and cytokine storm during infection [191]. Magnesium deficiency has also been linked to reduced immune response and consequently increased inflammation [192], and supplementation with vitamin D has been shown to increase immunity [193]. In addition, vitamin D in combination with NAC supplements has great potential in reducing oxidative stress as well as boosting immunity against SARS-CoV-2 infection [191].

### 6.7 Vitamin C

Vitamin C is a water-soluble essential vitamin, the deficiency of which leads to oxidative stress and inflammation, and reduced immunity. It has been reported that a normal plasma level is 50 μmol/L [194], with hypovitaminosis below ∼ 23 μmol/L and deficiency below 11 μmol/L [195]. Thus, patients with vitamin C deficiency may, in general, be at higher risk for COVID-19 and thus benefit from taking vitamin C. An observational study of 21 critically ill COVID-19 patients admitted to the ICU found that the surviving 11 patients had plasma levels of 29 μmol/L of vitamin C whereas the non-survivors had 15 μmol/L [196]. A recent clinical trial of vitamin C showed a potential benefit for critically ill patients with COVID-19 by improving oxygenation [197]. Vitamin C has been found to increase the production of IFNs which enhances the antiviral response [198,199]. Furthermore, vitamin C has been shown to reduce inflammation, even cytokine storm, by inactivating the NF-κB pathway [200]. It can also reduce the formation of neutrophil extracellular trap (NET) which is associated with vascular damage and blood clotting [201]. In addition, it reduces oxidative stress resulting in improved endothelial integrity and wound healing, which may be a potentially beneficial strategy for early and severe SARS-CoV-2 infections [202,203,204].

## 7 Conclusion

Growing evidence suggests that levels of antibody neutralization against Omicron infection decreased compared to ancestral strains of SARS-CoV-2 of those who received the primary vaccine or those who were previously infected with SARS-CoV-2. However, the neutralizing antibody levels have remained greater than 55% after primary vaccination and improved with booster doses to above 74%. In contrast to the finding of the humoral immune response, multiple datasets of cellular immunity concluded that 70–80% of CD4^+^ and CD8^+^ responses were maintained for Omicron infection, those who had previously been infected, and/or had been previously vaccinated. Well-preserved T cell immunity to Omicron can help in protecting against severe disease, and possibly reduce the risk of hospitalization and even death. In a true sense, due to the emergence of different variants of SARS-CoV-2, the vaccine alone cannot provide 100% protection against the pandemic. In addition to variant dependence, immunocompromised individuals have significantly reduced vaccine efficacy, even after a booster dose. Therapeutic interventions targeting oxidative stress, cytokine storm, and viral replication are expected to be effective in the management of severe or critical COVID-19 patients. Recent observational, preclinical and RCTs suggest that the Paxlovid™, Molnupiravir, Fluvoxamine, and Tempol have retained the antioxidants, anti-cytokine, and antiviral activity against Omicron-associated COVID-19 infection. Importantly, existing public health precautions such as wearing masks, avoiding enclosed spaces, maintaining physical distance, and hand hygiene that is effective against previous variants should be effective against the Omicron variant. In addition to these, we recommend getting at least 8 h of sleep each night, avoiding stress and fatigue, adequate physical exercise, adequate sunlight exposure, as well as taking micronutrients, including vitamins (vitamin D and vitamin C) and minerals (magnesium, zinc, selenium) which enhance overall immunity and will be beneficial in preventing Omicron infection. Collectively, the present insights point to potential research gaps and will aid in the development of new-generation COVID-19 vaccines and antiviral drugs to combat Omicron, its sublineages, or upcoming new variants of SARS-CoV-2.

## Author contribution statement

M.S. Alam is the sole author of the article.

## Funding statement

This research did not receive any specific grant from funding agencies in the public, commercial, or not-for-profit sectors.

## Data availability statement

Data included in article/supp. material/referenced in article

## Declaration of interest’s statement

The authors declare no conflict of interest.
